# Kernelized *k*-Local Hyperplane Distance Nearest-Neighbor Model for Predicting Cerebrovascular Disease in Patients With End-Stage Renal Disease

**DOI:** 10.3389/fnins.2021.773208

**Published:** 2021-10-25

**Authors:** Xiaobin Liu, Xiran Zhang, Yi Zhang, Yijie Ding, Weiwei Shan, Yiqing Huang, Liang Wang, Xiaoyi Guo

**Affiliations:** ^1^Department of Nephrology, The Affiliated Wuxi People’s Hospital of Nanjing Medical University, Wuxi, China; ^2^NHC Key Laboratory of Nuclear Medicine, Jiangsu Key Laboratory of Molecular Nuclear Medicine, Jiangsu Institute of Nuclear Medicine, Wuxi, China; ^3^Yangtze Delta Region Institute (Quzhou), University of Electronic Science and Technology of China, Quzhou, China; ^4^Institute of Fundamental and Frontier Sciences, University of Electronic Science and Technology of China, Chengdu, China

**Keywords:** cerebrovascular disease, end-stage renal disease, local hyperplane, klotho, FGF23

## Abstract

Detecting and treating cerebrovascular diseases are essential for the survival of patients with chronic kidney disease (CKD). Machine learning algorithms can be used to effectively predict stroke risk in patients with end-stage renal disease (ESRD). An imbalance in the amount of collected data associated with different risk levels can influence the classification task. Therefore, we propose the use of a kernelized *k*-local hyperplane nearest-neighbor model (KHKNN) for the effective prediction of stroke risk in patients with ESRD. We compared our proposed method with other conventional machine learning methods, which revealed that our method could effectively perform the task of classifying stroke risk.

## Introduction

Chronic kidney disease (CKD) has become a prominent disease affecting global health. According to existing research, the global incidence of CKD is approximately 8–16% and has been increasing yearly (Jha et al., 2013). Cerebrovascular diseases, such as stroke, represent major CKD complications that lead to neurological dysfunction and death, with negative impacts on prognosis in patients with CKD. Cerebral apoplexy, which is a primary cause of death among patients with CKD, refers to a series of adverse events, including cerebral ischemia, hypoxia, and cerebral dysfunction, caused by acute cerebral vascular rupture or acute cerebrovascular embolism (Kelly and Rothwell, 2020). A cohort study showed that CKD progression and a decline in the glomerular filtration rate increased the stroke risk among patients with CKD by nearly 40%, accompanied by a significant increase in the mortality rate (Toyoda and Ninomiya, 2014). Therefore, exploring the risk factors associated with stroke among the CKD population and identifying effective early interventions are necessary steps to reducing morbidity and mortality due to stroke.

Hypertension, diabetes, and dyslipidemia are traditional risk factors that contribute to the development of cerebrovascular diseases in patients with CKD. In addition, recent studies have revealed non-traditional risk factors, such as inflammation, oxidative stress, and CKD-mineral bone disease (CKD-MBD), that impact the occurrence and development of cerebrovascular diseases among patients with CKD. These non-traditional risk factors accelerate a series of pathological processes, such as cerebrovascular endothelial injury and sclerosis, in patients with CKD, leading to cerebrovascular calcification, further changing hemodynamics, and ultimately causing cerebrovascular events (Allen and Bayraktutan, 2009).

Our previous work identified abnormal FGF23 and Klotho levels, inflammatory status, and malnutrition were the unconventional risk factors for vascular calcification and CKD-MBD in patients with end-stage renal failure (Maraj et al., 2018). Using machine learning methods to analyze the risk factors of CKD-MBD in patients with end-stage renal failure, we have found that elevated serum FGF23 levels in patients with ESRD is an independent risk factor for abdominal aortic calcification (Liu et al., 2021). Recent studies have also identified FGF23 as an independent risk factor for cerebrovascular diseases in both CKD and non-CKD populations (Wright et al., 2016). The CHADS2 (congestive heart failure, hypertension, age = 75 years, diabetes mellitus, stroke) and CHA2DS2-VASc (congestive heart failure, hypertension, age ≥ 75 years, diabetes mellitus, stroke or transient ischemic attack vascular disease, age 65 to 74 years, sex category) scores are currently well-recognized methods for predicting the risk of stroke in patients with CKD (Hsu et al., 2020). Therefore, in this study, based on previous research findings, we used machine learning algorithms to develop models that explore the scientificity and veracity of both traditional and non-traditional risk factors combined with the CHADS2 stroke scoring tool and an abdominal aortic calcification scoring method for the prediction of stroke risk in patients with ESRD, which could help clinicians identify cerebrovascular disease and provide early interventions by assessing various risk factors, potentially delaying the occurrence and development of stroke, reducing morbidity and mortality, and improving prognosis among patients with ESRD.

## Materials and Methods

### Assessment of the CHADS2 and CHA2DS2-VASc Scores

We calculated the CHADS2 score based on the scoring system, as follows (de Bie et al., 2017): 1 point each was assigned for age ≥ 75 years, the presence of hypertension, diabetes mellitus, and congestive heart failure, and 2 points each were assigned for transient ischemic attack or a history of stroke. In addition, we calculated the CHA2DS2-VASc score based on the scoring system, as follows: 1 point each was assigned for congestive heart failure, hypertension, age between 65 and 74 years, diabetes mellitus, female sex, and vascular disease, whereas 2 points each were assigned for a history of stroke and age ≥ 75 years. CKD was defined as estimated glomerular filtration rate (eGFR) < 60 mL/min/m^2^ and classified as stages 3, 4, or 5 based on the eGFR level (30–59, 15–29, or <15 mL/min/1.73 m^2^, respectively) combined with kidney damage lasting for longer than 3 months. Patients were categorized into two groups according to their CHADS2 and CHA2DS2-VASc risk scores: (1) low-risk group (0–1 score) for CHADS2 and CHA2DS2-VASc scores and (2) high-risk group (≥2 scores) for CHADS2 and CHA2DS2-VASc scores.

### Calculation of the Nutritional Indexes

The Geriatric Nutrition Risk Index (GNRI) = [14.89 × serum albumin (g/dl)] + [41.7 × (actual body weight/ideal body weight)] (Yamada et al., 2020). Serum levels of intact FGF23, klotho, fetuin-A, and interleukin-6 were determined using two-site enzyme-linked immunosorbent assays (Elabscience Biotech, Wuhan, China).

### Abdominal Aortic Calcification Integration Method: Abdominal Aortic Calcification Score

All patients underwent lateral lumbar X-ray examinations within 1 week of biochemical blood examinations to assess abdominal aortic calcification corresponding to L1 to L4 (Asher et al., 2021). Each patient was scored based on the length of the calcified plaques identified on the anterior and posterior walls of the abdominal aorta, with each segment scored between 0 and 3 points, as follows: 0 points for no calcification; 1 point for calcification less than one-third of the arterial wall length; 2 points for calcification between one-third and two-thirds of the artery wall length; and 3 points if calcification covers more than two-thirds of the arterial wall length. Each lumbar segment is scored separately for both the posterior and anterior walls, resulting in a total score of 0–24 points. Table 1 shows the demographic and clinical details of our data set.

**TABLE 1:** Data set information.

No.	Feature	Value	r*
1	Sex (male/female)	32/27	–0.0455
2	Age (years)	55.83 ± 15.60	0.4010
3	Smoking (yes/no)	1/58	–0.0847
4	BMI (kg/m^2^)	23.56 ± 3.12	0.1639
5	DM (yes/no)	24/35	0.4847
6	CI (yes/no)	3/56	0.0025
7	CHD (yes/no)	5/54	0.0433
8	Systolic blood pressure (mmHg)	155.69 ± 23.63	–0.1150
9	Diastolic blood pressure (mmHg)	88.11 ± 13.71	–0.2043
10	Phosphate binder (yes/no)	36/23	–0.2141
11	Hemoglobin (g/L)	85.38 ± 18.12	–0.2584
12	C-reactive protein (mg/L)	11.74 ± 35.61	0.3016
13	Serum creatinine (μmol/L)	785.09 ± 368.62	–0.4252
14	Serum glucose (mmol/L)	5.48 ± 2.28	0.2608
15	Serum calcium (mmol/L)	2.08 ± 0.24	0.0520
16	Serum phosphorus (mmol/L)	1.81 ± 0.38	–0.0862
17	Total glyceride (mmol/L)	1.69 ± 1.08	–0.0542
18	Total cholesterol (mmol/L)	4.53 ± 1.42	–0.0466
19	Low density lipoprotein-C (mmol/L)	2.45 ± 0.96	–0.0252
20	High density lipoprotein-C (mmol/L)	0.97 ± 0.55	0.0866
21	HbA1c (%)	5.82 ± 1.03	0.2151
22	Serum albumin (g/L)	34.31 ± 6.61	–0.1308
23	25-OH Vitamin D3 (ng/ml)	7.86 ± 4.55	0.3850
24	iPTH (pg/ml)	274.50 ± 306.31	–0.0225
25	GNRI	96.06 ± 12.76	–0.0078
26	FGF23 (pg/ml)	32.21 ± 53.02	–0.0966
27	Klotho (ng/ml)	2.38 ± 2.33	0.0443
28	Interleukin-6 (pg/ml)	25.37 ± 53.69	0.2634
29	Fetuin-A (pg/ml)	3.0320e+05 ± 2.0606e+05	–0.0234
30	AACS	1.95 ± 1.55	0.2113
31	CHADS2 score	1.93 ± 1.11	0.4247
32	Group (low-risk group/high-risk group)	25/34	0.4619
33	CHA2DS2-VASc score	2.79 ± 1.50	0.5097

*Denotes each feature correlated with vascular calcification level using Pearson correlation coefficient (r).

*BMI, body mass index; DM, diabetes mellitus; CI, cerebral ischemia; CHD, coronary heart disease; HbA1c, glycated hemoglobin; iPTH, intact parathyroid hormone; GNRI, Geriatric Nutritional Risk Index; FGF23, fibroblast growth factor 23; AACS, Abdominal Aortic Calcification Score, CHADS2, congestive heart failure, hypertension, age = 75 years, diabetes mellitus, stroke; CHA2DS2-VASc, congestive heart failure, hypertension, age ≥ 75 years, diabetes mellitus, stroke or transient ischemic attack vascular disease, age 65–74 years, sex category.*

### The *k*-Local Hyperplane Distance Nearest-Neighbor Model

Vincent and Bengio developed an improved version of the *k*-nearest-neighbor algorithm (KNN), called the *k*-local hyperplane (LH) nearest-neighbor algorithm (HKNN) (Vincent and Bengio, 2002). The purpose of HKNN is to estimate the distance from the test sample in each class to its corresponding LH, which is built using the nearest k samples of the test sample. Suppose there are *C* classes; HKNN will obtain the predicted results of the test sample by calculating the minimum distance from *C* LHs. For the *c*-th class, the *c*-th LH is based on the nearest *k* neighbors of *x* in the training set, where *x* belongs to the *c*-th class. The *c*-th hyperplane is expressed as follows:


(1) $$L\,H_{k}^{c}=\left\{p^{c}|p^{c}={\bar{N}}^{c}+\sum_{i=1}^{k}\alpha_{i}^{c}\,V_{i}^{c},\alpha_{i}^{c}\in R^{k},i=1,\ldots ,k\right\}$$

where ${\bar{N}}^{c}=\frac{1}{k}\,\sum_{i=1}^{k}N_{i}^{c}$ is the centroid of the *k* neighbors of *x* in class *c*; $N_{i}^{c}$ is the *i*th neighbor of the test sample *x* in class *c*; and $V_{i}^{c}=N_{i}^{c}-{\bar{N}}^{c}$. The objective function of the test sample *x* to the *c*th LH is as follows:


(2) $${\left(L\,H_{k}^{c}\,\left(x\right)\right)}^{2}={||x-{\bar{N}}^{c}-\sum_{i=1}^{k}\alpha_{i}^{c}\,V_{i}^{c}||}^{2}+\lambda \,\sum_{i=1}^{k}{\left(\alpha_{i}^{c}\right)}^{2}$$

where λis the parameter of regular term. α^*c*^ can be calculated as:


(3) $$\left({\left(V^{c}\right)}^{T}\,V^{c}+\lambda \,I\right)\;\alpha^{c}={\left(V^{c}\right)}^{T}\,\left(x-{\bar{N}}^{c}\right)$$

where $\alpha^{c}={\left(\alpha_{1}^{c},\alpha_{2}^{c},\ldots ,\alpha_{k}^{c}\right)}^{T}$ and $V^{c}={\left(V_{1}^{c},V_{2}^{c},\ldots ,V_{k}^{c}\right)}_{d=k}$. The predictive result for test sample *x* is defined as:


(4) $$c\,l\,a\,s\,s\,c=m\,i\,n\,{||x-{\bar{N}}^{c}-\sum_{i=1}^{k}\alpha_{i}^{c}\,V_{i}^{c}||}^{2},c=1,2,\ldots ,C$$

To further improve the performance of the model, we applied feature mapping and the kernel trick to HKNN to obtain a kernelized HKNN model (KNKNN). Let *x* map to *f* by *ϕ*: *χ* → *F* and set $\bar{x}=x-{\bar{N}}^{c}$. Eq. 2 can then be rewritten, as follows:


(5) $${\left(L\,H_{k}^{c}\,\left(x\right)\right)}^{2}={||\phi \,\left(\bar{x}\right)-\sum_{i=1}^{k}\alpha_{i}^{c}\,\phi \,\left(V_{i}^{c}\right)||}^{2}+\lambda \,\sum_{i=1}^{k}{\left(\alpha_{i}^{c}\right)}^{2}$$

We obtained the differential of Eq. 5 as follows:


(6a) $$\left({\left(\phi \,\left(V^{c}\right)\right)}^{T}\,\phi \,\left(V^{c}\right)+\lambda \,I\right)\,\alpha^{c}={\left(\phi \,\left(V^{c}\right)\right)}^{T}\,\phi \,\left(\bar{x}\right)$$

(6b) $$\alpha^{c}={\left({\left(\phi \,\left(V^{c}\right)\right)}^{T}\,\phi \,\left(V^{c}\right)+\lambda \,I\right)}^{-1}\,{\left(\phi \,\left(V^{c}\right)\right)}^{T}\,\phi \,\left(\bar{x}\right)$$

(6c) $$\alpha^{c}={\left(K\,\left(V^{c},V^{c}\right)+\lambda \,I\right)}^{-1}\,K\,\left(V^{c},\bar{x}\right)$$

where *K*(*V^c^*, *V^c^*) ∈ *R*^*k* = *k*^ is a Gram matrix calculated by the radial basis function (RBF), and $K\,\left(V^{c},\bar{x}\right)\in R^{k=1}$ is a vector. The RBF is defined as:


(7) $$K\,\left(x_{i},x_{j}\right)=e\,x\,p\,\left(-\gamma {∥x_{i}-x_{j}∥}^{2}\right)$$

where γ is the Gaussian kernel bandwidth.

**FIGURE 1:**
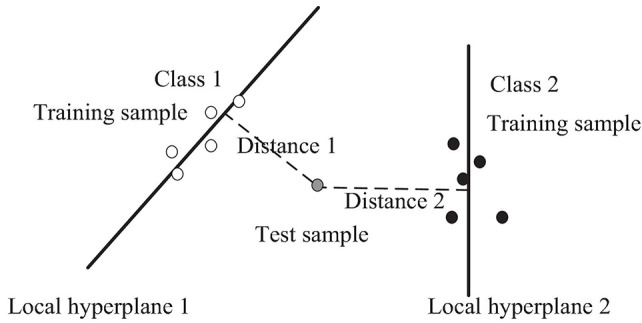
The schematic diagram of KNKNN.

To avoid overfitting of the model, KHKNN employed two strategies: (1) For test sample, KHKNN separately constructs a local hyperplane for each category by linear representation of neighborhood samples. It can alleviate the parameter (number) sensitivity of neighbors. (2) When constructing the hyperplane, we added the regular term (L2) of the coefficient. The schematic diagram of KNKNN is shown in Figure 1.

## Results

### Measurements

In this study, accuracy (ACC), sensitivity (SN), specificity (SP), positive predictive value (PE), negative predictive value (NPV), a weighted average of the PE and sensitivity (F_*score*_), and Matthews correlation coefficient (MCC) were calculated as follows:


(8a) $$A\,C\,C=\frac{T\,P+T\,N}{T\,P+F\,P+T\,N+F\,N}$$

(8b) $$S\,N=\frac{T\,P}{T\,P+F\,N}$$

(8c) $$S\,p\,e\,c=\frac{T\,N}{T\,N+F\,P}$$

(8d) $$P\,E=\frac{T\,P}{T\,P+F\,P}$$

(8e) $$N\,P\,V=\frac{T\,N}{T\,N+F\,N}$$

(8f) $$F_{s\,c\,o\,r\,e}=2\times \frac{S\,N\times P\,E}{S\,N+P\,E}$$

(8g) $$M\,C\,C=\frac{T\,P\times T\,N-F\,P\times F\,N}{\sqrt{\left(T\,P+F\,N\right)\times \left(T\,N+F\,P\right)\times \left(T\,P+F\,P\right)\times \left(T\,N+F\,N\right)}}$$

where *TP*, *TN*, *FN*, and *FP* are the number of true positive, true negative, false negative, and false positive results, respectively. The high-risk patients are positive samples, whereas the low-risk patients are negative samples.

### The Optimal Parameters

**FIGURE 2:**
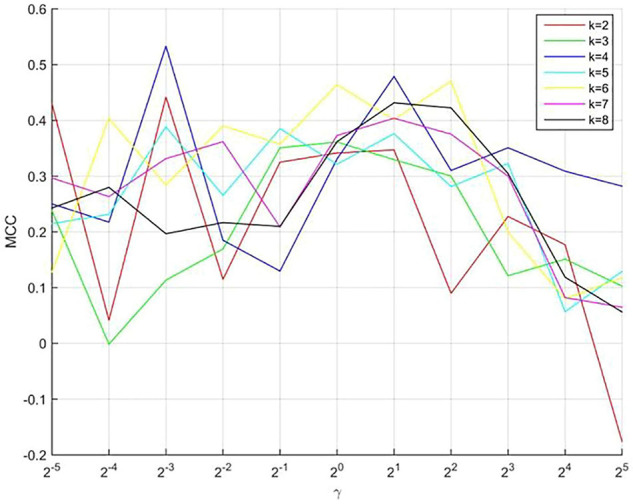
The predictive performance of the model using different parameters.

In our model, three parameters (*k*, γ, and λ) must be selected. To make the model robust, we set λ as 1. The optimal parameters of *k* and γ were selected using a grid search. The value of *k* ranges from 2 to 8 (maximum number of negative samples), with a step of 1. The value of γ ranges from 2^–5^ to 2^5^ with a step of 2^1^. The results are shown in Figure 2, which shows the predictive performance of the model using different parameters. When *k* and γare 4 and 2^–3^, respectively, the best MCC (0.5393) value was obtained.

### Comparison of Performance Between Our Method and Other Existing Methods

We compared the performance of several traditional classifiers with our method using the obtained dataset. The results are shown in Table 2. The compared classifiers included artificial neural network with backpropagation (ANN-BP), support vector machines (SVM), Takagi-Sugeno-Kang fuzzy system (TSK-FS), KNN, HKNN, and KHKNN. For small samples, KNN (ACC: 86.67%), HKNN (ACC: 85.00%), and TSK-FS (ACC: 78.33%) achieved good results. Our method (KHKNN) achieved the best MCC (0.5393) and ACC (89.67%) on small dataset. KHKNN was found to have an SN of 94.00%, with Spec reaching 60.00%. Our method achieved a relatively balanced performance for the recognition of both positive and negative samples.

**TABLE 2:** Comparison of performance between our method and other existing methods using the PDB1075 data set (Jackknife test evaluation).

Methods	MCC	ACC (%)	SN (%)	Spec (%)	PE (%)	NPV (%)	F_*score*_
ANN-BP	0.3521	77.58	82.18	**60.00**	92.32	40.00	0.8541
SVM	NaN	86.52	100	0	86.52	NaN	0.9272
TSK-FS	0.3803	78.33	82.91	**60.00**	92.00	43.33	0.8612
KNN	0.3697	86.67	**94.18**	40.00	90.73	50.00	0.9237
HKNN	0.4865	85.00	90.55	50.00	91.92	**66.67**	0.9094
KHKNN	**0.5393**	**89.67**	94.00	**60.00**	**94.52**	63.33	**0.9407**

*MCC, Matthews correlation coefficient; ACC, accuracy; SN, sensitivity; Spec, specificity; PE, positive predictive value; NPV, negative predictive value; F_score_, weighted average of the PE and sensitivity; ANN-BP, artificial neural network with backpropagation; SVM, support vector machines; TSK-FS, Takagi-Sugeno-Kang fuzzy system; KNN, k-nearest-neighbor; HKNN, k-local hyperplane distance nearest-neighbor algorithm; KHKNN, kernelized HKNN; NaN, not a number. The best results in each column are in bold faces.*

## Discussion

Stroke is one of the most serious complications among patients with CKD, leading to brain dysfunction and even death. Over the past 10 years, scholars have conducted a large number of mechanistic studies and epidemiological investigations exploring the kidney–brain interaction. The results of these studies have indicated that the kidney and brain have similar anatomical and functional characteristics. For example, both organs feature an arterial system that automatically adjusts perfusion pressure to ensure a continuous and relatively stable blood flow. In patients with CKD, cerebrovascular sclerosis occurs due to calcifications that form in the arterial system, disrupting the autoregulation function and allowing cerebrovascular events to occur (Lau et al., 2017).

The risk of stroke in patients with CKD is much higher than that in patients without CKD (Chen et al., 2012), and the stroke risk increases further as renal functional defects progress to ESRD. In recent years, in addition to traditional risk factors, such as hypertension, diabetes, and dyslipidemia, the influence of non-traditional risk factors on the occurrence of cerebrovascular calcification in patients with CKD has gained increasing attention, including inflammation, malnutrition, and the FGF23/klotho axis. The results of previous studies performed at our center have indicated that abnormal FGF23, klotho, and fetuin-A levels and malnutrition represent risk factors for abdominal aortic calcification in patients with ESRD (Maraj et al., 2018). FGF23 has been to play an important role in phosphate regulation. Klotho is the receptor protein for FGF23, which participates in regulating bone, calcium, and phosphorus metabolism; protecting the integrity of blood vessels; and inhibiting vascular calcification through the formation of FGF23-klotho complexes. The FGF23/klotho axis is a key participant in CKD-MBD and is closely related to vascular calcification and cerebrovascular diseases (Moldovan et al., 2014). Relevant studies have shown that an elevated FGF23 level is a risk factor for ischemia and hemorrhagic stroke in patients with CKD (Wright et al., 2014).

The CHADS2 and CHA2DS2-VASc scores are considered to be effective evaluation tools for predicting the risk of ischemic stroke in patients with CKD. Among patients with a high risk of ischemic cerebrovascular accidents, the administration of secondary prevention agents, such as anticoagulation and antithrombosis factors, when indicated by the cerebral ischemia score warning system, has been shown to greatly reduce the incidence of stroke and improve prognosis (Toyoda et al., 2014). The results of this study revealed a correlation between the CHADS2 and CHA2DS2-VASc scores in patients with ESRD and vascular calcification, which indirectly suggests the existence of an important relationship between vascular calcification and ischemic stroke. Moreover, when we combined the ischemic stroke scoring tool with traditional stroke risk factors, such as vascular calcification, to predict the risk of CKD stroke, the results were more reasonable, with a stronger scientific basis, than the use of the stroke scoring tool alone to predict risk.

The sample size is very small, so a simple machine learning model is preferred to solve the classification problem. Among them, KNN and SVM are suitable methods. The KNN algorithm is very sensitive to the number of neighboring samples. In the original feature space, the model cannot achieve satisfactory results of prediction. Therefore, we proposed KHKNN on the basis of KNN and HKNN to solve the above two problems. In the results section, KHKNN has obtained good prediction results. KHKNN separately constructs a local hyperplane for each category of test sample. The prediction result is determined by evaluating the distance (minimum) from the test sample to the hyperplane of each category. Therefore, it can alleviate the parameter sensitivity of KNN and avoid overfitting. The prediction result is determined by evaluating the distance (minimum) from the test sample to the hyperplane of each category.

In our study, KHKNN was employed to predict the risk of cerebrovascular disease among patients with ESRD. KHKNN estimates the distance from the test sample in each class to its corresponding LH in a high-dimensional feature space. Unlike KNN, KHKNN is not as sensitive to the parameter *k*, and its prediction performance is better than that of the ANN-BP and SVM models for small data sets. Compared with other models (ANN-BP, SVM, TSK-FS, KNN, and HKNN), our model achieved the best MCC (0.5393) and ACC (89.67%) values, showing that our method has good robustness and may be useful for determining clinical risk in the future.

## Conclusion

We proposed a KHKNN method to filter noise samples, improve the generalization ability of the model, and obtain good results. Although our method achieves a relatively balanced performance for the recognition of positive and negative samples, the following disadvantages must be acknowledged. (1) The sample size must be further increased to minimize prediction bias. (2) No detailed analysis was performed to examine the contribution of various patient factors. (3) Although the kernel function was used to map the original space to further improve the performance, the interpretability of the model was affected. Fuzzy systems will be introduced in the future to improve interpretability. At present, artificial intelligence technology has been used for large-scale medical information processing (Jian et al., 2019; Guo et al., 2021; Jiang et al., 2021a, b; Zhang et al., 2021a, b) and bioinformatics (Qian et al., 2021; Zou et al., 2021) on a large scale, with good performance. (4) The k-dimensional tree is employed to speed up the search speed of the nearest neighbor samples. In addition, parallel computing technology also can increase the speed of searching. In the future, we will use artificial intelligence methods to solve additional clinical problems.

## Data Availability Statement

The original contributions presented in the study are included in the article/supplementary material, further inquiries can be directed to the corresponding author/s.

## Ethics Statement

The studies involving human participants were reviewed and approved by the Human Ethics Committee (Wuxi People’s Hospital Ethics Committee, No. KS2019041). The patients/participants provided their written informed consent to participate in this study.

## Author Contributions

XL: methodology, data curation, and writing – original draft preparation. XZ: methodology and writing – original draft preparation. WS and XG: methodology and data curation. YD: methodology and software. LW: methodology, supervision, and writing – reviewing and editing. All authors contributed to the article and approved the submitted version.

## Conflict of Interest

The authors declare that the research was conducted in the absence of any commercial or financial relationships that could be construed as a potential conflict of interest.

## Publisher’s Note

All claims expressed in this article are solely those of the authors and do not necessarily represent those of their affiliated organizations, or those of the publisher, the editors and the reviewers. Any product that may be evaluated in this article, or claim that may be made by its manufacturer, is not guaranteed or endorsed by the publisher.
